# Systematic analysis and functional characterization of the chitinase gene family in *Fagopyrum tataricum* under salt stress

**DOI:** 10.1186/s12870-024-05971-z

**Published:** 2024-12-20

**Authors:** Qingqing Li, Yongyi Yang, Xue Bai, Lun Xie, Suzhen Niu, Biao Xiong

**Affiliations:** 1 College of Tea/Agrobioengineering Sciences, Key Laboratory of Plant Resource Conservation and Germplasm Innovation in Mountainous Region (Ministry of Education), Guizhou University, Guiyang, 550025 China; 2 College of Forestry, Guizhou University, Guiyang, 550025 China; 3 Department of Botany, University of British Columbia, Vancouver, V6T 1Z4 Canada

**Keywords:** *CHIs* family, *F. tataricum*, Salt stress, Expression pattern, Subcellular localization, Transient expression

## Abstract

**Background:**

Chitinases (CHIs) are glycosidases that degrade chitin, playing critical roles in plant responses to both abiotic and biotic stress. Despite their importance, the *CHI* family’s systematic analysis and evolutionary pattern in *F. tataricum* (Tartary buckwheat) yet to be explored.

**Results:**

This study analyzed their phylogenetic relationships, conserved motifs, gene structures, syntenic relationships, physiological functions, and biochemical properties. This research identified 26 *FtCHIs* and examined their expression patterns under different salt stress conditions and across various tissues. Differential expression analysis revealed a significant upregulation of multiple *FtCHIs* in response to salt stress, which RT-qPCR further validated. Additionally, subcellular localization experiments demonstrated that Ft_chitinaseIV-2 is localized in vacuoles. The results of transient·transformation showed that·overexpression of *Ft_chitinaseIV-2* could·enhance the salt tolerance of plants.

**Conclusions:**

The findings provide new insights into the role of *CHI*s in stress tolerance and lay the groundwork for future research on the functional characterization of *FtCHIs*. Understanding the molecular mechanisms of CHI-mediated stress responses could contribute to developing stress-resistant crops.

**Supplementary Information:**

The online version contains supplementary material available at 10.1186/s12870-024-05971-z.

## Background

Chitinase proteins are found across various biological systems, playing essential roles in modifying or degrading chitin. In organisms such as fungi, insects, and crustaceans, chitinase primarily functions to modify chitin, a key component of their cell walls. In bacteria, chitinases degrade chitin to provide carbon and nitrogen nutrition [1, 2]. In plants, chitinases are involved in responses to both biotic and abiotic stresses, as well as in growth and developmental processes [3–5]. These enzymes are classified into two prominent families, glycosyl hydrolase family 18 (GH18) and glycosyl hydrolase family 19 (GH19), based on their catalytic domains, which contain approximately 220–230 amino acid (aa) residues [6, 7]. These families are divided into several classes, each with distinct structural and functional features [8, 9]. For example, GH18 includes classes III and V, found across various organisms, while GH19, comprising classes I, II, and IV, is mainly present in plants [10, 11]. Variations in domain structure among these classes suggest diverse functional roles, ranging from stress response to developmental regulation.

Plant chitinases are also recognized as pathogenesis-related (PR) proteins. Although plants do not contain chitin, their chitinase levels increase rapidly in response to stress from fungi, bacteria, or insects, inhibiting the growth and reproduction of these pathogens by targeting their chitin-containing cell walls. Overexpression of chitinase genes has been shown to enhance resistance to various pathogens. For instance, the overexpression of *LOC_Os11g47510* in rice improves resistance to *Rhizoctonia solani* [12]. Similarly, transgenic expression of chitinase genes from balsam pear (*Momordica charantia L)* enhances resistance to the fungal pathogen *Phytophthora nicotianae* in transgenic tobacco and *Verticillium wilt* in transgenic cotton [13]. *ClCHIs* plays a key role in the resistance of watermelon to *Fusarium oxysporum* infection [14]. In addition, examples of endogenous or heterologous expression of chitinase genes to enhance plant resistance to fungi or bacteria have also been found in tobacco [15], bananas [16], tomatoes [17], maize[ [18]]. tea [19], poplar and other plants [20]. These findings highlight the critical role of chitinases in enhancing plant resistance to pathogens.

Beyond pathogen defense, chitinases also play significant roles in plant growth, development, and responses to hormones and abiotic stresses. For instance, in watermelon, the expression of chitinase genes changes significantly under drought, salt, and low-temperature stress[ [14]]. Mutations in chitinase genes can lead to altered plant morphology and stress responses, as seen in Arabidopsis thaliana (*A. thaliana*), where class II chitinase mutations cause abnormal cell wall formation and changes in plant growth [21]. Research shows that *CaCHIIV1* plays an essential role in the defense mechanism of pepper plants against *Phytophthora capsici* infection and response to drought stress [22]. In potatoes, *StuCHI26* and *StuCHI8* had a solid response to salt stress, and after salicylic acid treatment, the expression levels of the four *StuCHIs* were higher than those under salt stress [23]. Chitinase gene expression can also be influenced by plant hormones and various stress conditions, such as salicylic acid, jasmonic acid, ethylene, mechanical damage, heavy metals, and ozone exposure [13, 24–30]. These multifaceted roles underline the importance of chitinases in plant biology.

Tartary buckwheat (*Fagopyrum tataricum*) is a medicinal and edible crop with high economic and nutritional value [31]. However, it faces significant challenges from abiotic stresses, including cold, drought, heat, and salt stress [32–35]. Despite the known roles of chitinases in stress responses and plant development, their functions in Tartary buckwheat remain unexplored.

To address this gap, we conducted a comprehensive genome-wide identification of chitinase genes in Tartary buckwheat, identifying 26 *FtCHI* members. We examined their chromosome location, phylogenetic relationship, gene structures, motif characteristics, expression patterns in different tissues, and responses to salt stress. We also explored the subcellular localization of these proteins. These findings provide a foundation for further research into the functional roles of *FtCHIs*, potentially enhancing Tartary buckwheat’s resilience to environmental stresses.

## Results

### Identification of *FtCHI* gene family members and their physicochemical properties

A total of the 26 *FtCHI* genes were identified in the *F. tataricum* genome based on BLASTP and HMMER searches and further confirmed using the CDD and SMART databases. The *FtCHI* family was classified into four classes based on conserved domains and motifs. Class III and V contain the GH18 domain, while class II and class IV contain the GH19 domain. The *FtCHIs* accordingly named as *Ft_chitinaseII-1* ∼ *Ft_chitinaseV-9*. The physicochemical property predictions revealed significant variation in the length and molecular weight of FtCHIs. The protein length ranged from 92 aa (Ft_chitinaseIV-6) to 648 aa (Ft_chitinaseV-5), and molecular weight ranged from 10.21 kDa (Ft_chitinaseIV-6) to 55.61 kDa (Ft_chitinaseIV-4). The predicted pI varied between 4.31 (Ft_chitinaseIV-6) and 9.60 (Ft_chitinaseIV-11), with 11 FtCHIs exhibiting alkaline pI values (> 7), suggesting a potential role in alkaline environments, while 15 FtCHIs had acidic pI values (< 7). Subcellular localization predictions indicated that FtCHIs are localized in diverse cellular compartments, including the cell wall, vacuole, extracellular, cell membrane, chloroplast and nucleus (Table 1).

### Phylogenetic analysis and multiple sequence comparisons of *FtCHIs*

**Fig. 1:**
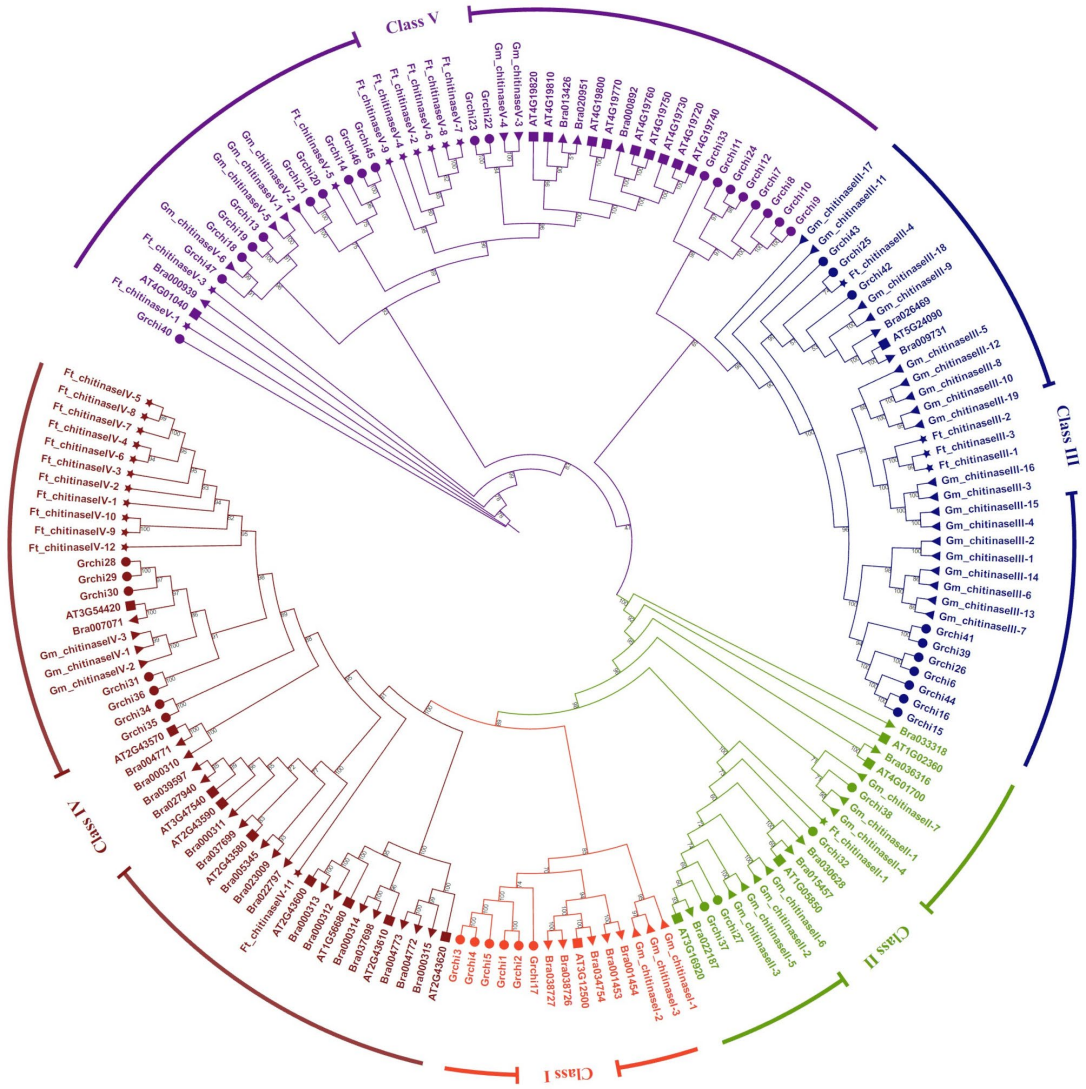
Phylogenetic tree of chitinase genes from *F. tataricum*(Ft), *(A) thaliana* (AT), *(B) rapa*(Bra), *G. raimondii*(Gr) and *G. max*(Gm). The phylogenetic tree was built using the maximum likelihood (ML) method by IQ-TREE, and the resulting phylogenetic tree was visualized and edited using iTOL. The roman numerals (I-V) representing each gene cluster, genes from each species were labeled with different colors and the number represents the bootstrap support

Phylogenetic analysis of 169 chitinase protein sequences, conducted using the ML method, identified five significant classes of chitinase proteins (Fig. 1). Using the classification of ATCHI as a reference, FtCHIs were categorized into four distinct groups. The GH18 subfamily includes class II and class V, with class II containing a single member and class V comprising nine members. The GH19 subfamily includes classes III and IV, with class III containing four members and class IV containing 12 members. This classification provides insights into the evolutionary relationships among the FtCHI family.

**Fig. 2:**
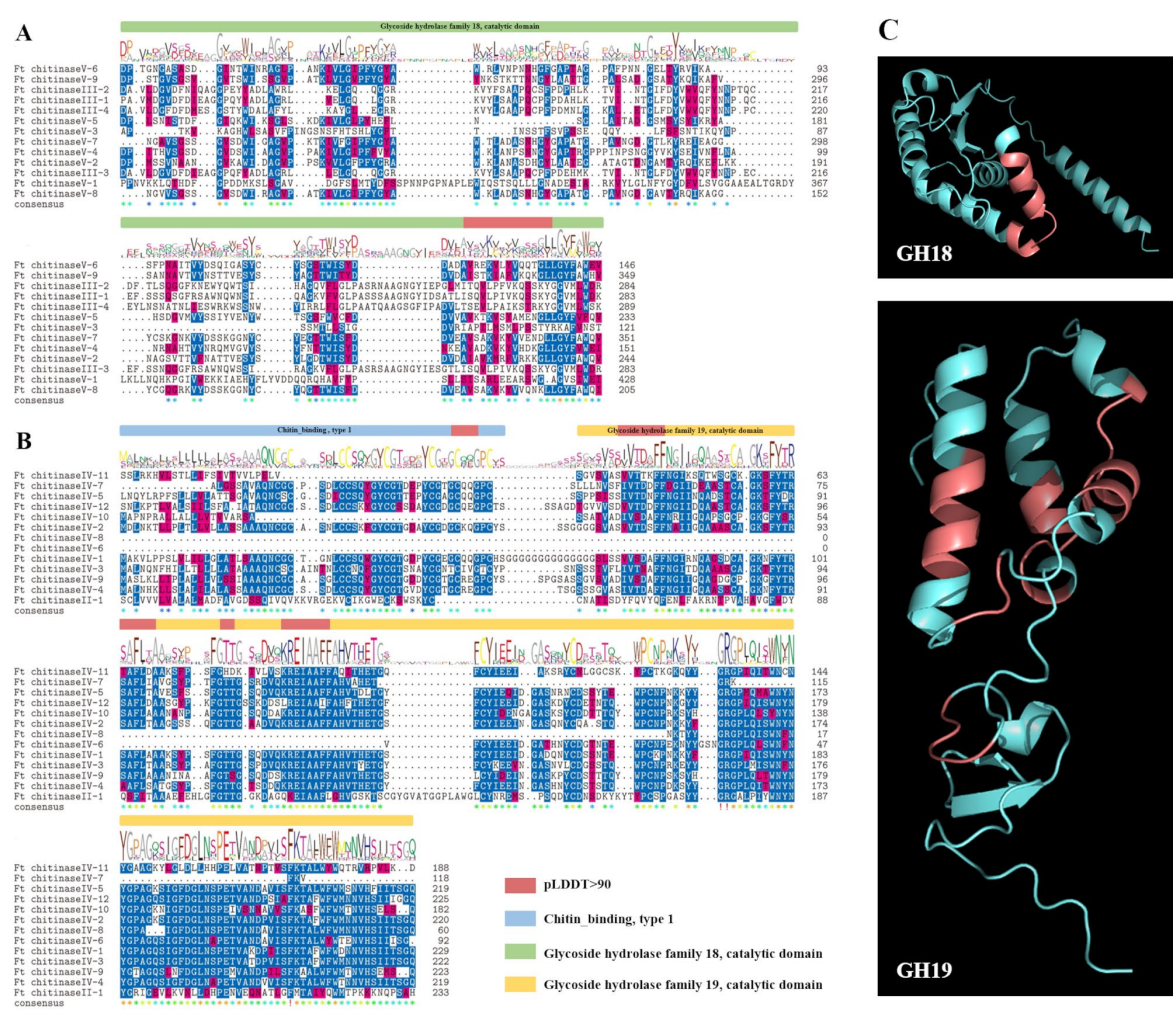
Multiple sequence comparisons of FtCHIs. (**A** and **B**) Multiple sequence alignment of GH18 and GH19, green boxes indicate structures with Glycoside hydrolase family 18 catalytic domain, yellow boxes indicate structures with Glycoside hydrolase family 19 catalytic domain, and blue boxes indicate structures with Chitin_binding, type 1. (**C**) three-dimensional structure prediction of FtCHIs. Red color indicates structures with pLDDT > 90

Multiple sequence comparisons of chitinase amino acids from *F. tataricum* indicated that most of the FtCHI sequences contained catalytic domain: Glycoside hydrolase family 18 catalytic domain, Glycoside hydrolase family 18 catalytic domain and chitin_binding type 1 (Fig. 2A). Some FtCHIs do not have chitin_binding domains, for example, Ft_chitinaseII-1, Ft_chitinaseIV-6, Ft_chitinaseIV-8, Ft_chitinaseIV-10, and Ft_chitinaseIV-11 (Fig. 2B). In addition, alphafold2 prediction showed that there is one conserved site in the FtCHIs of GH18 subgroup, and its structure is helix-loop-helix. There are five conserved sites in GH19 with the structures: loop; loop; helix; helix-loop-helix; helix-loop-helix (Fig. 2C).

**Table 1:** CHI gene family in F. tataricum

Gene Name	Gene ID	Class	Gene Position		Amino acid	MW (kDa)	pI	Domains	Predicted Subcellular Localization
			Start	End					
Ft_chitinaseII-1	FtPinG0000874100.01.T01	II	60,803,265	60,805,450	322	35.38	6.96	GH19	Vacuole
Ft_chitinaseIII-1	FtPinG0002342600.01.T01	III	441,305	442,240	299	31.92	6.27	GH18	Extracellular
Ft_chitinaseIII-2	FtPinG0002342700.01.T01	III	437,441	438,454	300	32.92	8.80	GH18	Extracellular
Ft_chitinaseIII-3	FtPinG0002343000.01.T01	III	435,451	436,414	299	32.02	5.55	GH18	Vacuole
Ft_chitinaseIII-4	FtPinG0006963100.01.T01	III	6,928,599	6,929,904	305	33.45	8.76	GH18	Vacuole
Ft_chitinaseIV-1	FtPinG0004180700.01.T01	IV	57,162,230	57,163,246	275	28.79	5.77	GH19	Vacuole
Ft_chitinaseIV-2	FtPinG0004180900.01.T01	IV	57,158,491	57,159,971	266	28.00	7.86	GH19	Vacuole
Ft_chitinaseIV-3	FtPinG0004181100.01.T01	IV	57,155,328	57,156,678	265	28.5	6.77	GH19	Extracellular; Vacuole
Ft_chitinaseIV-4	FtPinG0004181300.01.T01	IV	57,128,896	57,143,165	526	55.61	5.11	GH19	Vacuole
Ft_chitinaseIV-5	FtPinG0004181500.01.T01	IV	57,122,861	57,123,818	280	29.90	4.44	GH19	Vacuole
Ft_chitinaseIV-6	FtPinG0004181700.01.T01	IV	57,121,250	57,121,528	92	10.21	4.31	GH19	Cell wall
Ft_chitinaseIV-7	FtPinG0004181900.01.T01	IV	57,117,712	57,118,068	118	12.48	5.13	GH19	Cell wall
Ft_chitinaseIV-8	FtPinG0004182100.01.T01	IV	57,116,094	57,116,455	118	12.73	4.57	GH19	Cell wall
Ft_chitinaseIV-9	FtPinG0006445900.01.T01	IV	17,149,335	17,150,479	269	28.11	5.07	GH19	Vacuole
Ft_chitinaseIV-10	FtPinG0006446100.01.T01	IV	17,152,591	17,153,396	228	24.13	7.57	GH19	Extracellular
Ft_chitinaseIV-11	FtPinG0007187600.01.T01	IV	5,510,898	5,512,275	234	25.68	9.60	GH19	Extracellular
Ft_chitinaseIV-12	FtPinG0009560900.01.T01	IV	50,545,438	50,547,067	271	28.70	4.60	GH19	Vacuole
Ft_chitinaseV-1	FtPinG0000327200.01.T01	V	49,664,024	49,667,948	439	49.86	7.80	GH18	Cell wall
Ft_chitinaseV-2	FtPinG0000828100.01.T01	V	59,310,424	59,311,470	262	28.71	9.06	GH18	Cell wall
Ft_chitinaseV-3	FtPinG0000952800.01.T01	V	11,488,629	11,489,608	145	16.27	9.16	GH18	Cell membrane
Ft_chitinaseV-4	FtPinG0001842300.01.T01	V	26,730,777	26,731,328	168	18.24	6.82	GH18	Cell wall
Ft_chitinaseV-5	FtPinG0003642700.01.T01	V	52,634,115	52,636,760	648	74.36	7.54	GH18	Chloroplast; Nucleus
Ft_chitinaseV-6	FtPinG0007078000.01.T01	V	12,890,507	12,891,004	165	17.77	4.76	GH18	Cell wall
Ft_chitinaseV-7	FtPinG0008844200.01.T01	V	54,319,218	54,320,651	368	39.42	4.42	GH18	Cell wall
Ft_chitinaseV-8	FtPinG0008844600.01.T01	V	54,323,622	54,325,058	222	23.49	8.40	GH18	Cell wall
Ft_chitinaseV-9	FtPinG0008936400.01.T01	V	41,726,236	41,727,345	365	39.27	9.02	GH18	Cell wall

### Conserved motif and gene structure analysis of *FtCHIs*

**Fig. 3:**
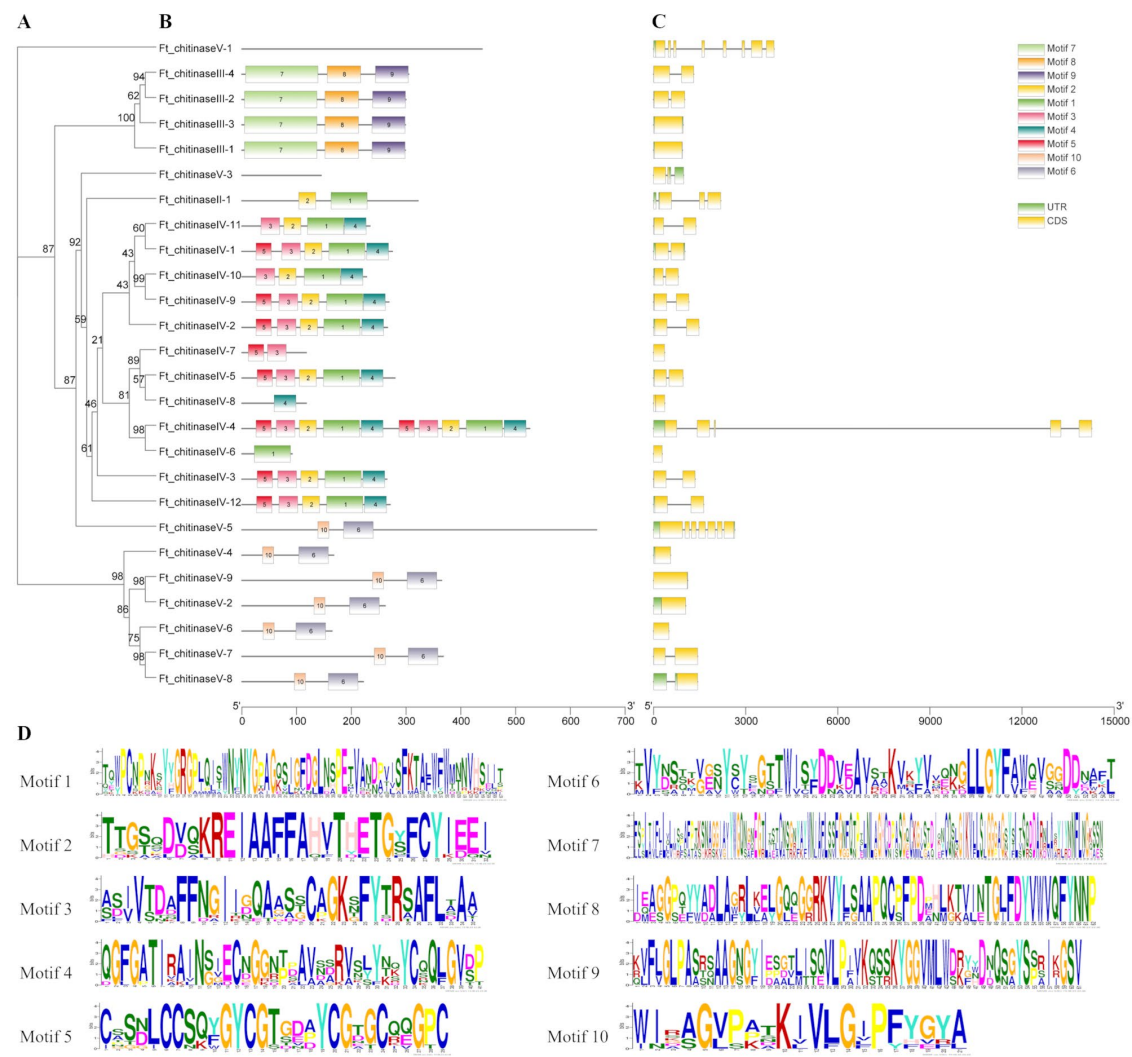
Phylogenetic relationships, gene structures and conserved motifs of the *FtCHI* family. (**A**) Phylogenetic tree of *FtCHIs*, the phylogenetic tree was constructed using the IQ-TREE software through the Maximum Likelihood method with 1000 bootstrap replicates, the number indicates the bootstrap support. (**B**) Conserved motifs of *FtCHIs*, different motifs are represented by specific colors. (**C**) Gene structure of *FtCHI*s. Untranslated regions, introns, and exons are each denoted by a green box, black line, and yellow box, respectively. (**D**) Conserved motif logos of *FtCHIs*

The exon-intron organization was examined using the MEME online tool to further investigate the structural diversity of *FtCHIs*. Significant structural differences were observed across subfamilies (Fig. 3C). The 26 *FtCHIs* contained 10 conserved motifs (Motif 1 to10), ranging from 21 to 133 aa in length (Fig. 3B). Notably, *Ft_chitinaseV-1* and *Ft_chitinaseV-3* needed to have identified conserved motifs but were found to contain the GH18 domain via the NCBI CD-Search tool. Class III contains motifs 7, 8, and 9, suggesting potential functional similarity. Motifs 6 and 10 were found in class V, while class II had motifs 1 and 2. Additionally, motif 5 was exclusively identified in class IV. Within class IV, all members except *Ft_chitinaseIV-6* and *Ft_chitinaseIV-8* contained motifs 1 to 4, highlighting their conserved structure.

### *Cis*-acting elements in *FtCHI* promoters

**Fig. 4:**
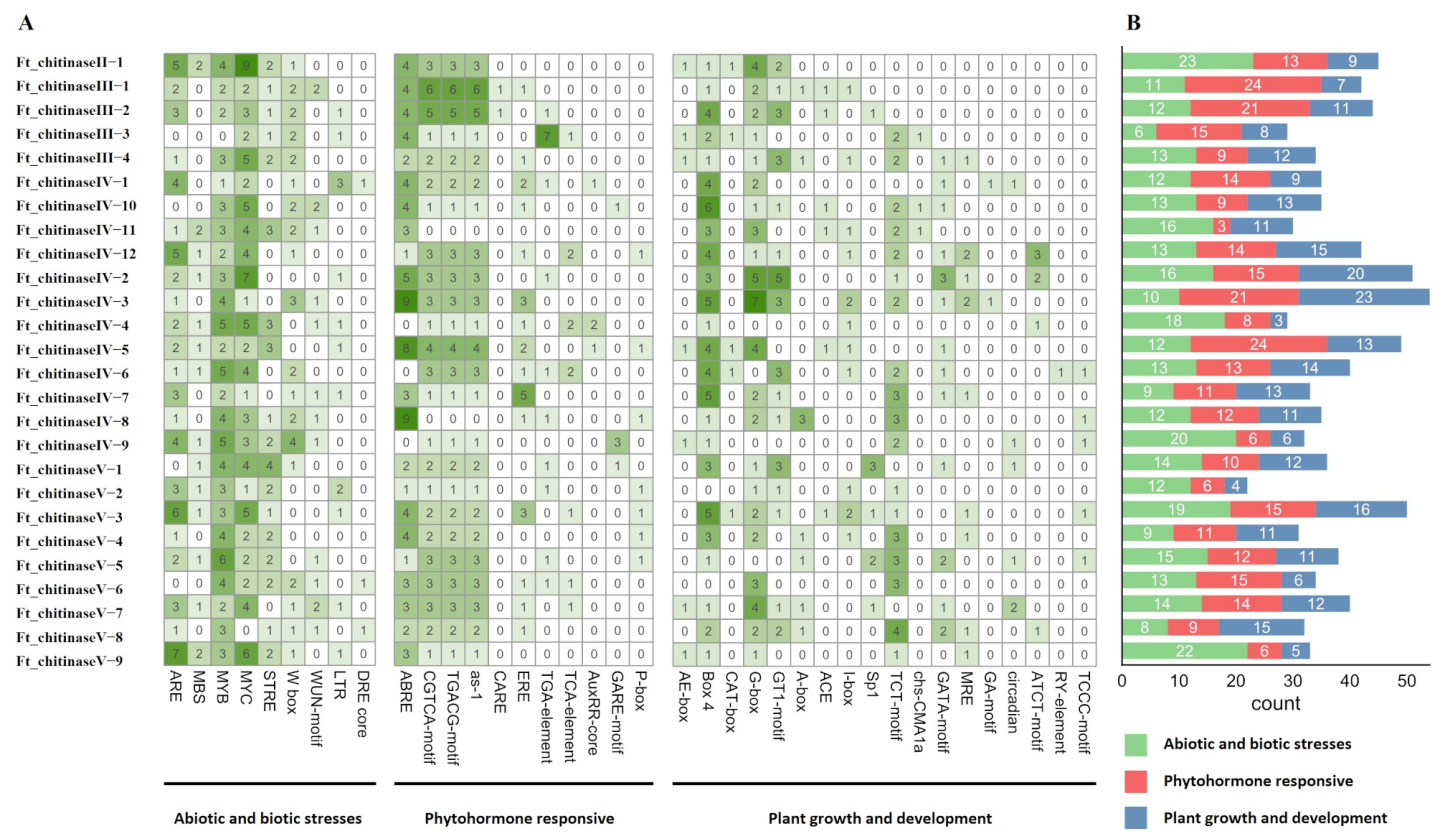
Predicted cis-acting regulatory elements in *FtCHI* promoters. Promoter regions 2000 bp upstream of the CHI genes translation start sites were analyzed by PlantCARE. (**A**) the different intensity colors and numbers of the grid indicate the numbers of different promoter elements in the *FtCHI* genes. The 38 types of cis-acting elements are divided into three categories. (**B**) the different colored histogram represents the sum of the cis-acting elements in each category

The 2000 bp promoter regions upstream of *FtCHIs* initiation transcription start sites were analyzed using the PlantCARE database. Thirty-eight *cis*-acting elements were identified (Fig. 4A), with all *FtCHIs* promoters containing the core CAAT and TATA boxes essential for promoter and enhancer activity, respectively. Additionally, nine abiotic and biotic stress-responsive elements, 11 phytohormone-responsive elements, and 18 plant growth and development *cis-*acting elements were detected. Notably, the DRE core element, associated with drought and high-salinity stress, was identified in *Ft_chitinaseIV-1*, *Ft_chitinaseV-6*, and *Ft_chitinaseV-8*. Furthermore, *Ft_chitinaseIII-1* contained 24 phytohormone-responsive elements, and *Ft_chitinase IV-3* 23 plant growth and development-related elements (Fig. 4B). The diversity of *cis*-acting elements suggests that *FtCHIs* may be involved in various biological processes, including responses to abiotic stresses, light, hormones, meristem expression, and seed development.

### Chromosomal distribution and collinearity analysis

**Fig. 5:**
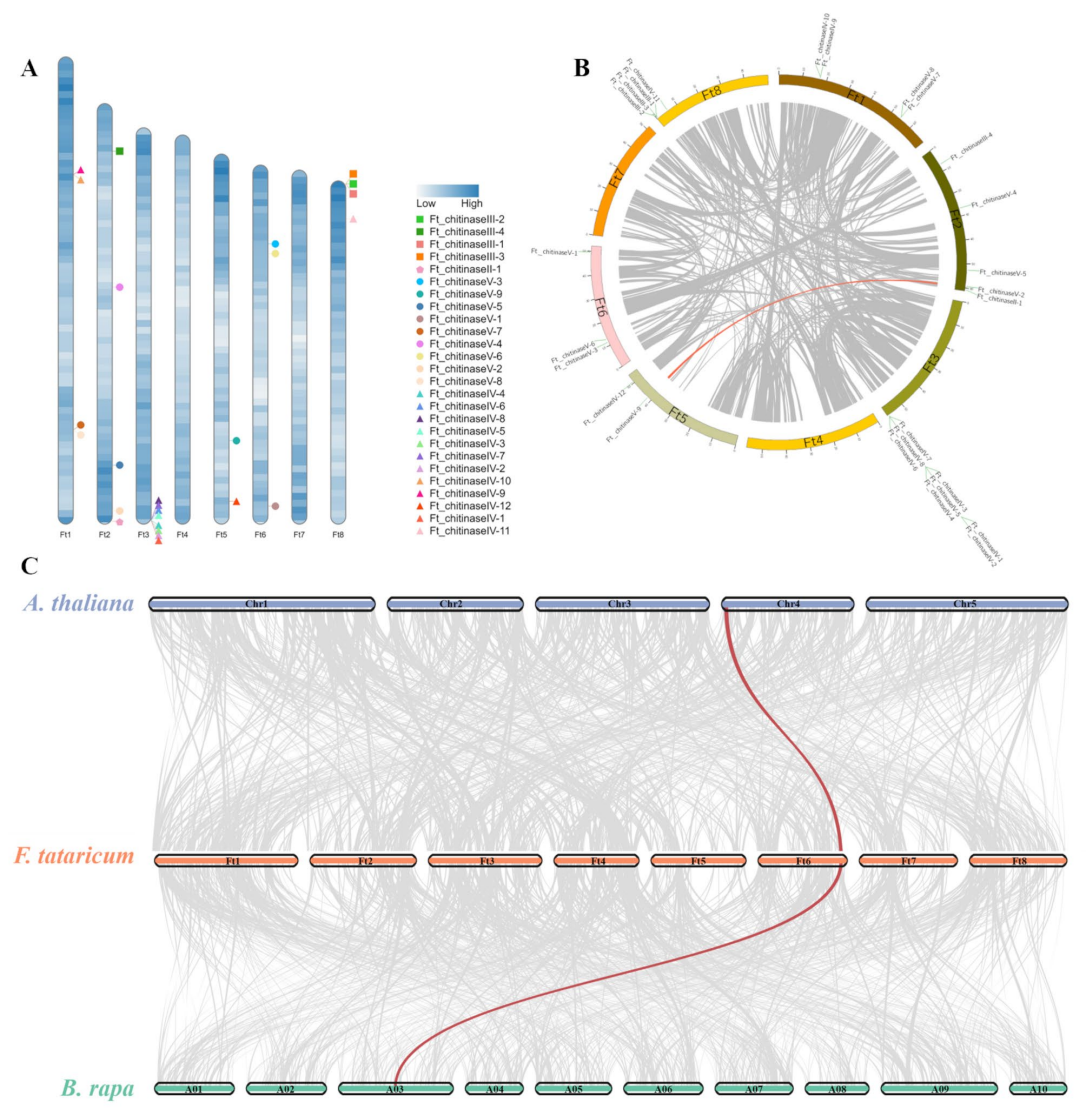
Chromosomal distribution and collinearity analysis of the *FtCHI* genes. (**A**) Different symbol represent different family class, and different colors represent different genes. (**B**) Collinearity analysis of *FtCHIs* in *F. tataricum* genome. All of the genome’s collinear blocks are shown by gray lines, while gene pairs with duplication events are represented by red lines. (**C**) Collinearity analysis of CHIs among *F. tataricum*, *(A) thaliana*, and *(B) rapa*. *FtCHIs*, *AtCHIs*, and *BraCHIs* are represented by orange, blue, and cyan, respectively. The gray lines in the background represent the collinear blocks identified in the genomes of *F. tataricum*, *(A) thaliana*, and *(B) rapa*. Red lines indicate the *CHI* gene pairs

Based on genomic annotation data, 26 *FtCHIs* were mapped to chromosomes (Fig. 5A). The distribution analysis showed that *FtCHIs* are unevenly dispersed across the genome. Chromosomes 2, 3, 5 and 6 each contained 5, 8, 2, 3 genes, while chromosomes 1and 8 contained four. Most genes were predominantly localized at the ends of chromosomes, indicating non-uniform distribution patterns.

Collinearity analysis was performed to explore the evolutionary relationships among 26 *FtCHIs* (Fig. 5B). The results revealed two segmentally duplicated genes, *Ft_chitinaseV-2* and *Ft_chitinaseV-9*, in 26 *FtCHIs*, located on chromosomes 2 and 5, respectively. Comparative collinearity analysis (Fig. 5C) across *F. tataricum*, *(A) thaliana*, and *(B) rapa* revealed that *F. tataricum* shared one collinear gene, *Ft_chitinaseV-1*, with *(A) thaliana* (*AT4G01040*) and *(B) rapa* (*Bra000939*), suggesting substantial evolutionary divergence. Among *FtCHIs*, *Ft_chitinaseV-1* had the highest frequency of collinear occurrences, and its sequence alignment with *AT4G01040* indicated high homology, suggesting potential functional similarities.

### Differential expression levels of *FtCHI* genes in different plant tissues

**Fig. 6:**
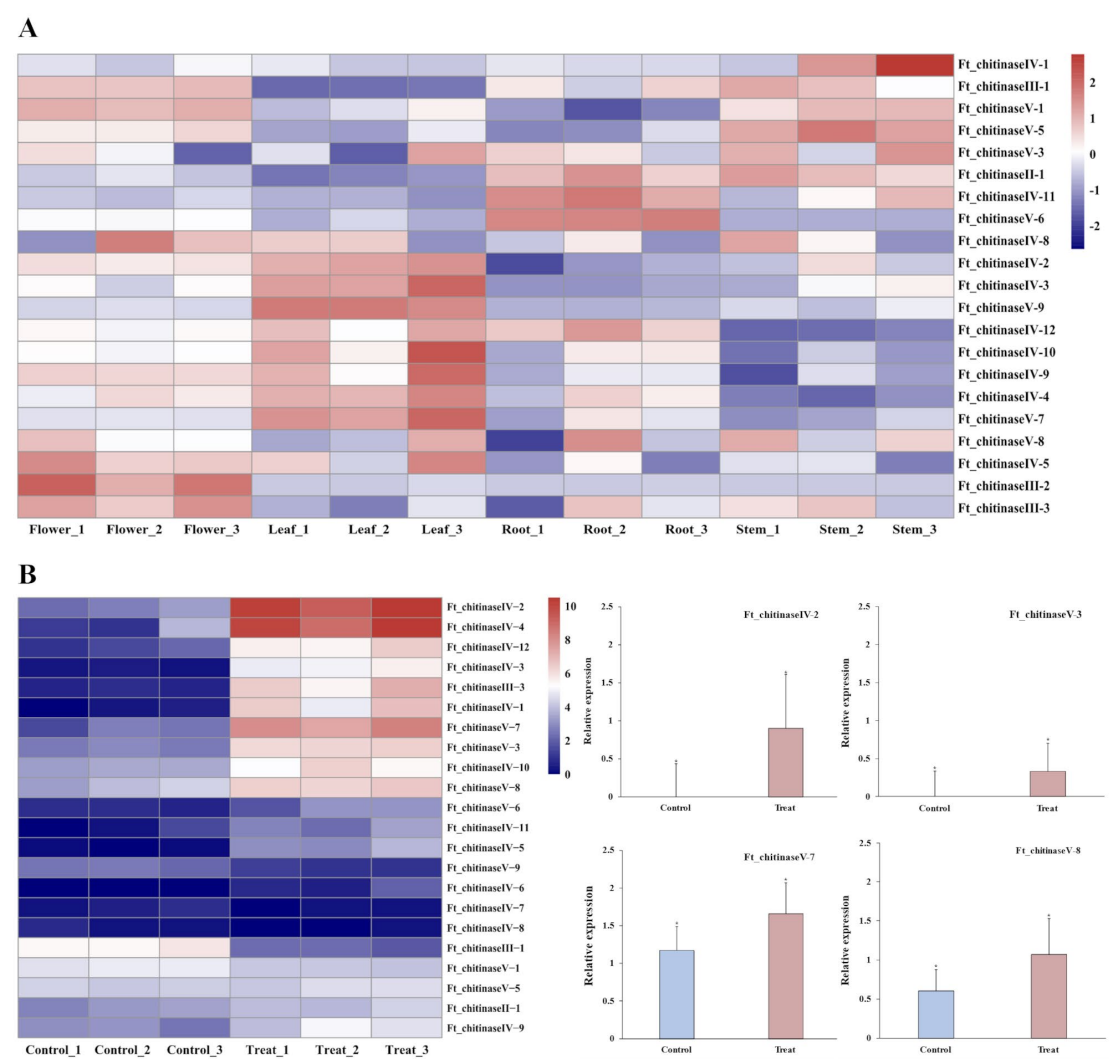
The expression patterns of *FtCHI* genes in different plant tissues salt stress treatment. (**A**) Tissue-specific expression analysis of 26 *FtCHI* family genes in different *F. tataricum* tissues (roots, stems, leaves, flowers), the color scale represents the relative transcript levels of *FtCHIs*, with blue denoting down-regulation and red denoting up-regulation. (**B**) The expression patterns of FtCHI genes during salt stress treatment. After salt stress treatment, RNA was extracted from the leaves of *F. tataricum* after 3 h (Control) and 12 h (Treat) treatment. The transcript levels of 4 *FtCHIs* were determined by qRT-PCR using *FtH*_*3*_ as an endogenous control, and were visualized by R packages

RNA-seq data were analyzed to evaluate the expression patterns of *FtCHIs* across different tissues, including roots, stems, leaves, flowers *F. tataricum* (Fig. 6A). Several genes exhibited tissue-specific expression: *Ft_chitinaseIV-5*, *Ft_chitinaseIII-2* and *Ft_chitinaseIII-3* were primarily expressed in flowers, while *Ft_chitinaseIV-2*, *Ft_chitinaseIV-3*, *Ft_chitinaseIV-4*, *Ft_chitinaseV-7*, and *Ft_chitinaseV-9* had elevated expression in leaves. *Ft_chitinaseII-1*, *Ft_chitinaseIV-11*, and *Ft_chitinaseV-6* were strongly expressed in roots, and *Ft_chitinaseIV-1* showed higher expression in stems. Notably, *Ft_chitinaseIII-1*, *Ft_chitinaseV-1*, and *Ft_chitinaseV-5* exhibited high expression levels in both stems and flowers, while *Ft_chitinaseV-3* was predominantly expressed in roots and stems. These findings suggest functional differentiation among the *FtCHI* family members in different plant tissues.

### Differential expression levels of *FtCHI* genes in salt stress

Transcriptome data revealed distinct expression patterns of the *FtCHI* family members under salt stress, with significant differences between control (3 h) and treated (12 h) plants (Fig. 6B). Fifteen *FtCHIs*, including *Ft_chitinaseII-1*, F*t_chitinaseIII-2*, *Ft_chitinaseIII-4*, *Ft_chitinaseIV-5*, *Ft_chitinaseIV-6*, *Ft_chitinaseIV-7*, *Ft_chitinaseIV-8*, *Ft_chitinaseIV-9*, *Ft_chitinaseIV-11*, *Ft_chitinaseV-1*, *Ft_chitinaseV-2*, *Ft_chitinaseV-4*, *Ft_chitinaseV-5*, *Ft_chitinaseV-6*, and *Ft_chitinaseV-9* showed either low or no expression across all treatments. Among the other members, *Ft_chitinaseIII-1* was significantly down-regulated under salt stress, with *Ft_chitinaseIV-2* and *Ft_chitinaseIV-4* displaying the highest relative expression levels.

### Subcellular localization

**Fig. 7:**
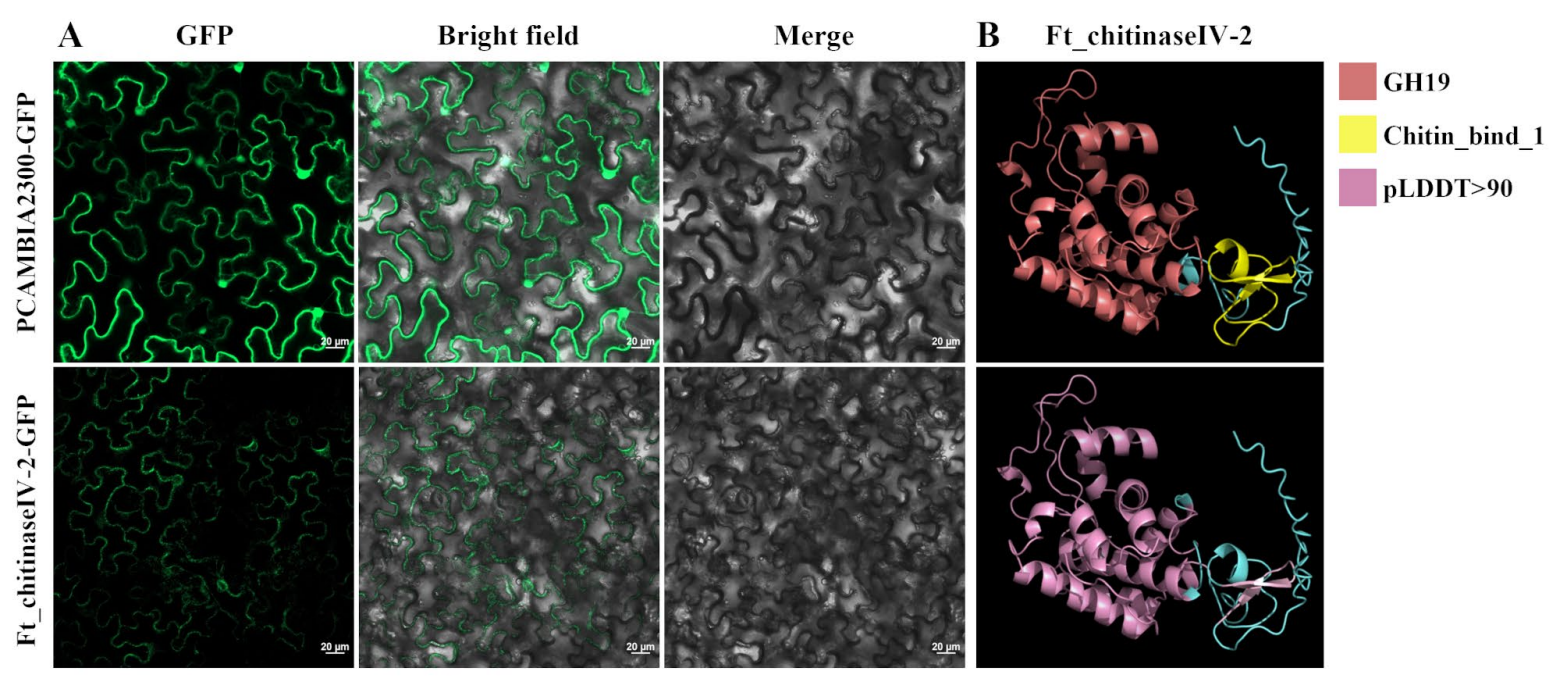
Subcellular localization and three-dimensional prediction of Ft_chitinaseIV-2. (**A**) Ft_chitinaseIV-2 is from transiently transformed *N. benthamiana* leaves and located in vacuole (scale bars = 20 μm). (**B**) FtCHI protein three-dimensional prediction. Red represents the GH19 structural domain, yellow represents the Chitin_bind_1, and blue represents amino acid sequence

Subcellular localization predictions indicated that Ft_chitinaseIV-2 was targeted to the vacuole. This finding was confirmed experimentally through transient expression of a Ft_chitinaseIV-2-GFP fusion protein in *N. benthamiana* leaves. After 48 h of incubation, fluorescence microscope revealed that the GFP signal was confined to vacuoles, corroborating the predicted vacuolar localization and suggesting a specific functional role for Ft_chitinaseIV-2 in this organelle (Fig. 7).

### Stress resistance function of *Ft_chitinaseIV-2*

**Fig. 8:**
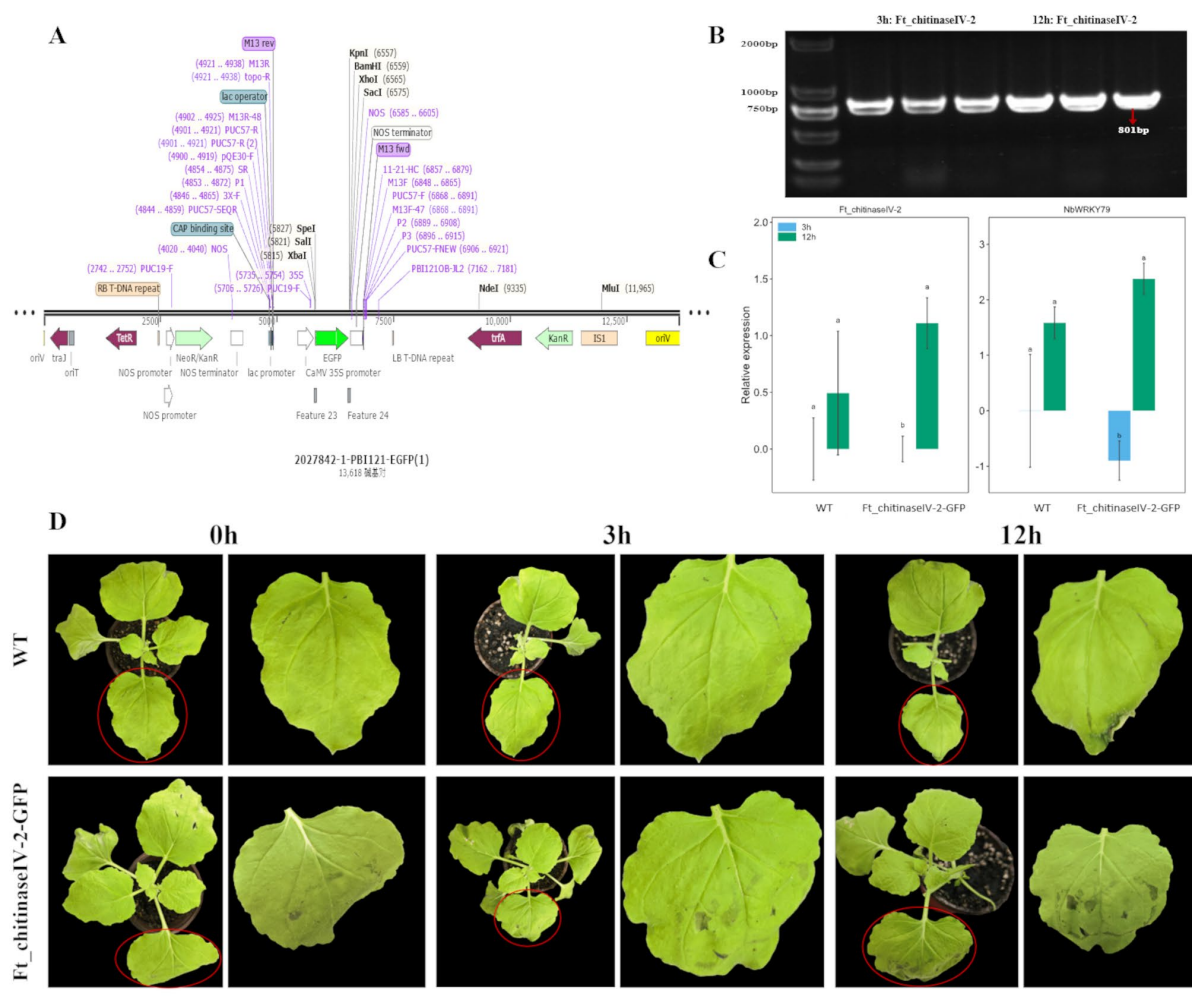
Gene expressions in the wild-type and *Ft_chitinaseIV-2*-overexpressing lines. (**A**) PCAMBIA2300-GFP plasmid mapping. (**B**) Electropherogram of *Ft_chitinaseIV-2*-overexpressing lines. (**C**) Gene expression levels in the leaves of the wild-type plants and *Ft_chitinaseIV-2* transgenic lines were determined under normal condition and salt treatment with 150 mM NaCl. The gene expression levels in the wild-type plants and transgenic lines during the salt stress responses as determined using RT-qPCR. Nbβ-actin were used as internal controls. The different letters above the columns indicate significant differences (*P* < 0.05) according to Duncan’s multiple range test. (**D**) Observation of salt stress phenotypes

To further investigate the stress resistance function of *Ft_chitinaseIV-2*, an overexpression vector Ft_chitinaseIV-2-GFP was transiently transformed into *N. benthamiana*, with wild-type plants serving as control. Under salt stress conditions, the expression of *Ft_chitinaseIV-2* was significantly up-regulated in the overexpressed plants compared to controls. Leaf samples from both transgenic and wild-type plants were analyzed, showing that while wild-type plants exhibited marginal leaf drying post-treatment, transgenic plants displayed only minor wilting (Fig. 8D). Additionally, gene expression levels in transgenic plants increased significantly over 12 h, with overexpressed plants maintaining higher levels than controls (Fig. 8C). These results suggest that Ft_chitinaseIV-2 enhances salt tolerance in tobacco, contributing to the mitigation of stress-induced damage. 

## Discussion

Tartary buckwheat is well-known for its high flavonoids content including rutin, which contributes to its medicinal and nutritional value, such as reducing hyperlipidemia, hypertension, and hyperglycemia. This makes it an essential crop for food and medicinal purposes, with increasing consumer demand due to its unique health benefits [36]. Chitinase, a glycoside hydrolase prevalent found in various plant species, plays critical roles in plant defense against pathogens and pests, growth regulation, and response to abiotic stresses. The *CHI* family has been identified in several plants, showing varying gene numbers across species-ranging from 24 in *A. thaliana* to 37 in rice, 159 in wheat, and 92 in tetraploid cotton [36–38]. However, no comprehensive study on the *CHI* family in *F. tataricum* has been reported until now.

This study identified 26 *FtCHIs* in *F. tataricum* through BLAST and domain identification. Chromosome localization revealed that *FtCHIs* are distributed across six chromosomes, with an uneven pattern unrelated to chromosome length. Phylogenetic analysis classified the *FtCHI* family into four distinct subgroups, two of which belonged to GH19 (class II, IV) and two to GH18 (class III, V). Notably, no members were detected in subgroup I, suggesting a possible gene contraction event during evolution. Structural predictions using Alphafold2 confirmed conserved structural domains within subgroups, while some members lacked chitin_binding_1 domain, hinting at possible gene mutations or rearrangements. Further, GH19 family members exhibited more conserved functional sites, suggesting their potential significance in growth and stress responses.

Motif and gene structure analysis showed conserved motifs specific to subgroups, such as motif 7, unique to subgroup III, and motifs 8 and 9 absent in other groups. Motif and gene structure analysis showed conserved motifs specific to subgroups, such as motif 7, unique to subgroup III, and motifs 8 and 9 absent in other groups. These patterns provide insights into the evolutionary relationships within the CHI gene family, with conserved motifs indicating functional correlation while distinct motifs suggesting functional differentiation. Expression pattern analysis of the *FtCHIs* across different tissues confirmed tissue-specific expression, a characteristic trait of *CHI* families. For instance, Ft_chitinaseIII-2 was predominantly expressed in flowers, while Ft_chitinaseV-9 exhibited enhanced expression in leaves, indicating possible specialization of these genes at different growth stages. These patterns provide insights into the evolutionary relationships within the *CHI* family, with conserved motifs indicating functional correlation while distinct motifs suggesting functional differentiation.

Previous studies have indicated tissue-specific expression of *CHIs* in plants, such as *Solanum tuberosum*, where *StuCHI20* is highly expressed in callus and tubers, while *StuCHI26* is restricted to roots, stolons, petioles and carpels [23]. Our expression analysis revealed that *Ft_chitinaseIII-2* is highly expressed in flowers, while *Ft_chitinaseV-9* is dominant in leaves, indicating tissue-specific functions of *FtCHIs*. Furthermore, studies have shown that leaf growth and expansion rates are influenced by environmental stress, such as drought, which significantly affects the development of leaf morphology, as observed in the *YABBY* family in *Camellia sinensis* [39]. Specifically, *CsFILa* expression decreased by 60-80% under drought stress, suggesting that such stress-related downregulation may play a significant role in controlling growth and stress resistance.

The *cis*-element analysis demonstrated the presence of regulatory elements responsive to abiotic stress (drought, cold, and salt), hormones (ABA, MeJA, and GA), and plant development in FtCHI promoters, suggesting that FtCHIs might play roles in stress responses. Notably, Ft_chitinaseV-8 harbored a salt-stress response element (DRE core) and was upregulated under salt stress. In contrast, Ft_chitinaseIV-2, lacking such an element, still showed increased expression under salt stress, indicating that gene regulation involves other pathways beyond *cis*-elements.

Subcellular localization experiments confirmed Ft_chitinaseIV-2’s localization in the vacuole, a compartment that plays a vital role in maintaining cellular homeostasis under salt stress by storing ions like Na^+^ [40]. Our transient expression experiments in *N. benthamiana* showed that *Ft_chitinaseIV-2* expression increased under salt stress, enhancing salt tolerance by regulating the expression of salt-resistance-related genes such as *NbWRKY79*. Finally, the study provides a prediction of the functional roles of each identified *FtCHI* gene under salt stress (Table 2). Genes such as *Ft_chitinaseIV-2* demonstrate significant roles in vacuolar sequestration of ions, contributing to enhanced salt stress tolerance. *Ft_chitinaseIII-1* shows reduced expression with prolonged salt exposure, indicating a potential regulatory feedback mechanism in the stress response. The expression patterns and subcellular localization suggest functional differentiation among FtCHI family members, providing insights into their diverse roles under salt stress conditions. These findings provide new insights into the molecular mechanisms underlying stress responses in *F. tataricum*.

**Table 2:** Comprehensive overview of FtCHI gene functions prediction in Fagopyrum tataricum under salt stress

FtCHI Gene	Subfamily	Expression Pattern Under Salt Stress	Subcellular Localization Prediction	Associated Regulatory Pathways	Proposed Function Predictin
*Ft_chitinaseIV-2*	GH19	Up-regulated, high expression	Vacuole	*FtWRKY79* up-regulation	Enhances salt tolerance viaion sequestration in vacuole [41]
*Ft_chitinaseV-9*	GH18	Up-regulated in leaves	Cell wall	Response to ABA and MeJA signals [42]	Involved in stress signal transduction.
*Ft_chitinaseIII-1*	GH18	Down-regulated with prolonged stress	Extracellular	Unknown	Potential negative regulator of stress response.
*Ft_chitinaseIII-2*	GH18	High expression in flowers	Extracellular	Light-responsive elements present	Supports growth and developmental processes.
*Ft_chitinaseV-8*	GH18	Up-regulated under salt stress	Cell wall	DRE core element involvement	Participates in drought and salt stress responses.

## Conclusion

Here, 26 *FtCHIs* were identified in *F. tataricum*, and their phylogenetic relationships, gene structures, conserved motifs, chromosomal distribution, and expression patterns were systematically analyzed. These genes are unevenly distributed on six chromosomes, and phylogenetic analysis divided them into four subgroups. Expression pattern analyses revealed that *FtCHIs* are involved in various aspects of *F. tataricum* growth, development, and stress responses, particularly in response to salt stress. Subcellular localization experiments confirmed that Ft_chitinaseIV-2 is localized in vacuoles, and transient transformation experiments demonstrated that overexpression of Ft_chitinaseIV-2 enhanced the expression of the salt-resistance *NbWRKY79*, thus improving stress resistance. The study lays the groundwork for further research on *FtCHIs* and their roles in stress resistance, offering valuable genetic resources for the molecular breeding of *F. tataricum*.

## Materials and methods

### Plant materials, growth conditions, and stress treatments

Tartary buckwheat (*F. tataricum*) seeds were soaked for 15 min, and any floating seeds were discarded. The selected seeds were sown in a petri dish with humid filter paper and grown in hydroponics at 22 °C for 14 days under 16/8 (light/dark) and relative humidity of approximately 60%. To assess the effects of salt stress, plants in similar growth stages were subjected to 150 mmol/L NaCl stress treatment. Cotyledons samples were collected at 3 and 12 h post-treatment, immediately frozen in liquid nitrogen, and stored at -80℃, total samples had three biological replicates. For subcellular localization studies, *Nicotiana benthamiana* (tobacco) plants were grown in pots under controlled conditions at 25 °C, with 16-hour light and 8-hour dark photoperiod and relative humidity of approximately 70%. The materials used in the experiment were from the Key Laboratory of Agricultural Bioengineering, Guizhou University.

### Identification of *FtCHI* members

The *F. tataricum* genome sequence was retrieved from the Tartary buckwheat Genome Project (TBGP; http://www.mbkbase.org/Pinku1/, accessed on 10 June 2023). *A. thaliana* Proteins sequences from *A. thaliana* were downloaded from the Arabidopsis Information Resource (TAIR, https://www.arabidopsis.org, accessed on 10 June 2023). To identify chitinase genes in *F. tataricum*, a BLASTP search was performed using 24 AtCHI sequences against *F. tataricum* genome. The GH18 (PF00704) and GH19 (PF00182) domains were downloaded from the Pfam database (https://www.ebi.ac.uk/interpro/entry/pfam, accessed on 10 June 2023) protein family databases and used in a HMMER search (version 3.3.2), with an E-value threshold of < 1e^−^ [43, 44]. The identified sequences were further verified for the presence of conserved domains using the NCBI-The Conserved Domain Database (https://www.ncbi.nlm.nih.gov/Structure/cdd/wrpsb.cgi, accessed on 28 July 2023) and SMART(http://smart.embl-heidelberg.de/, accessed on 28 July 2023). Proteins containing GH18 or GH19 to determine whether it belonged to the chitinase gene family. Molecular weight (MW) and theoretical isoelectric point (pI) of the identified proteins were calculated using the ExPASy (http://www.expasy.org/, accessed on 13 April 2023) compute pI/Mw tool. Subcellular localization was predicted using the Cell-PLoc 2.0 web server (http://www.csbio.sjtu.edu.cn/bioinf/plant-multi/, accessed on 13 April 2023). The tertiary structure of the identified proteins was predicted alphafold2 (https://colab.research.google.com/github/sokrypton/ColabFold/blob/main/AlphaFold2.ipynb, accessed on 1 April 2024).

### Phylogenetic analysis and multiple sequence comparisons of *FtCHIs*

Multiple sequences alignments of CHIs in *A. thaliana*, *Brassica rapa* (*B. rapa*), *Gossypium raimondii* (*G. raimondii*) and *Glycine max* (*G. max*) (Lv et al. 2022) were performed using MUSCLE program with default parameters [25, 37, 45–47]. Phylogenetic analysis was constructed using the maximum likelihood (ML) method in IQ-TREE, with bootstrap analysis with 1000 replicates to assess branch support. The resulting phylogenetic tree was visualized and edited using iTOL (https://itol.embl.de) [48, 49].

Multiple sequence alignments of FtCHIs were performed using MUSCLE, and domain information was verified through Interpro (https://www.ebi.ac.uk/interpro/search/sequence/, accessed on 21 May 2024). Subsequently, the FtCHIs protein sequences were uploaded to AlphaFold2 (https://colab.research.google.com/github/sokrypton/ColabFold/blob/main/AlphaFold2.ipynb, accessed on 27 May 2024) for three-dimensional structure prediction. Sequences with a predicted local distance difference test (pLDDT) score greater than 90 were considered to approximate the true structure. To identify conserved sites, amino acid sequences with pLDDT > 90 were extracted and subjected to further multiple sequence alignment. This approach allowed for the identification of conserved regions, which were used to infer potential functional sites within the FtCHIs proteins.

### Conserved motif and gene structure analysis of *FtCHIs*

*FtCHIs* coding sequences (CDS) were analyzed for gene structure using Gene Structure Display Server 2.0 (http://gsds.cbi.pku.edu.cn/). The amino acid sequences were submitted to the MEME suite server (http://meme-suite.org/) to identify conserved motifs with the following parameters: a maximum 10 conserved motifs and a motif width of 10 to 50 amino acids (aa). Gene structures were visualized using *Amazing Optional Gene Viewer* in TBtools. The promoter regions (∼ 2000 bp upstream) of *FtCHIs* were analyzed for cis-elements using PlantCARE (http://bioinformatics.psb.ugent.be/webtools/plantcare/, accessed on 10 November 2023) [50]. 

### Chromosomal distribution and collinearity analysis

The chromosomal location of *FtCHIs* was determined using the gene ID annotation from the *F. tataricum* genome, and the chromosome localization analysis was performed using the R package *RIdeogram*. Intraspecies and interspecies collinearity relationships were analyzed using MCScanX-2019 software, with results visualized in Circos 0.69 [51, 52].

### Salt stress and tissue-specific expression of *FtCHIs*

Transcriptomic data for *F. tataricum* under salt stress and from different tissues (flowers, roots, leaves, stems) were obtained from NCBI (https://www.ncbi.nlm.nih.gov/sra/, accessed on 9 August 2023) under accession number PRJNA522429 and PRJNA528524. After normalization, expression profiles were visualized as heatmaps using the R package *pheatmap*.

### Real time quantitative reverse transcription PCR (RT-qPCR) analysis

Total RNA was extracted from plant tissues using the RNAprep Pure Kit (DP441, TIANGEN, Beijing, China). The integrity of the RNA was verified via 1.0% agarose gel electrophoresis, and RNA purity and concentration were measured using the NanoPho-tometer N50 (Implen, Munich, Gemany). RNA was reverse transcribed using PrimeScriptTM RT Master Mix (TaKaRa, Tokyo), with the following conditions: 37 ℃ for 15 min and 85 ℃ for 5 s. Specific primers of 4 *FtCHIs* and internal controls *FtHis* (HM628903) were designed using NCBI Primer-BLAST (accessed on 10 August 2023). Primers were synthesized by TSINGKE Biotechnology Co., Ltd. (Beijing, China).

RT-qPCR was performed using the CFX96 real-time PCR system (Bio-RAD, Hercules, CA, USA), with a reaction volume of 25 µL containing 12.5 µL TB Green^®^ Premix Ex Taq™ II, 1 each of upstream and downstream primers, 2 µL cDNA, and 8.5 µL sterile distilled water. The cycling conditions were 95 ℃ for 30 s, followed by 39 cycles at 95 ℃ for 5 s and 60 ℃ for 30 s. Gene expression levels were calculated using the 2^−∆∆CT^ method with three biological replicates. Data analysis was performed using R 4.3.1.

### Subcellular localization

The coding sequences of Ft_chitinaseIV-2 and Ft_chitinaseV-8, without stop codons, were cloned into the PCAMBIA2300-GFP vector to create Ft_chitinaseIV-2-GFP and Ft_chitinaseV-8-GFP fusion proteins. Transient expression was performed in tobacco leaves, and fluorescence was detected 72 h post-infiltration using a Zeiss LSM900 confocal microscope, with excitation at 488 nm and emission detection between 550 and 650 nm for GFP.

### Transient expression of *Ft_chitinaseIV-2* in tobacco and salt stress response

The *Ft_chitinaseIV-2-GFP* vectors were transiently transformed into 4-week *N. benthamiana* plants using Agrobacterium-mediated transformation. After transformation, the tobaccos were subjected to 150 mmol/L NaCl for 3 and 12 h. Leaf samples from both transiently overexpressing *Ft_chitinaseIV-2* and wild-type tobaccos (WT) were collected for expression analysis of *Ft_chitinaseIV-2* and salt stress-responsive *NbWRKY79* via RT-qPCR to hypothesize the functional role of *Ft_chitinaseIV-2* under salt stress [53].

## Electronic Supplementary Material

Below is the link to the electronic supplementary material.

Additional file 1: Figure S1: The original and unprocessed gel image

Additional file 2: Table S1: The sequence of *FtCHIs*

Additional file 3: Table S2: Prediction of cis-regulatory elements in promoter regions of *FtCHIs*

Additional file 4: Table S3: The primer sequences in salt stress of *F. tataricum*

Additional file 5: Table S4: The primer sequences in salt stress of *N. benthamiana*

Additional file 6: Table S5: The results of genes for qRT-PCR

Additional file 7: Table S6: The results of genes for qRT-PCR

## Data Availability

The transcriptome data used in this article are available for download from NCBI (https://www.ncbi.nlm.nih.gov/sra/) under accession number PRJNA522429 and PRJNA528524. The sequence information of CHI family genes in F. tataricum, (A) thaliana, (B) rapa, G. raimondii and G. max was collected from the TBGP (http://www.mbkbase.org/Pinku1/), TAIR (https://www.arabidopsis.org), and and Phytozome v13 (https://phytozome-next.jgi.doe.gov/). All other datasets supporting the results of this article are included within the article and its additional files.
