# Supplementary figures and images for: Systematic analysis and functional characterization of the chitinase gene family in Fagopyrum tataricum under salt stress
Source: BMC Plant Biol. 2024 Dec 20;24:1222. doi: 10.1186/s12870-024-05971-z (PMC11660572; doi:10.1186/s12870-024-05971-z)

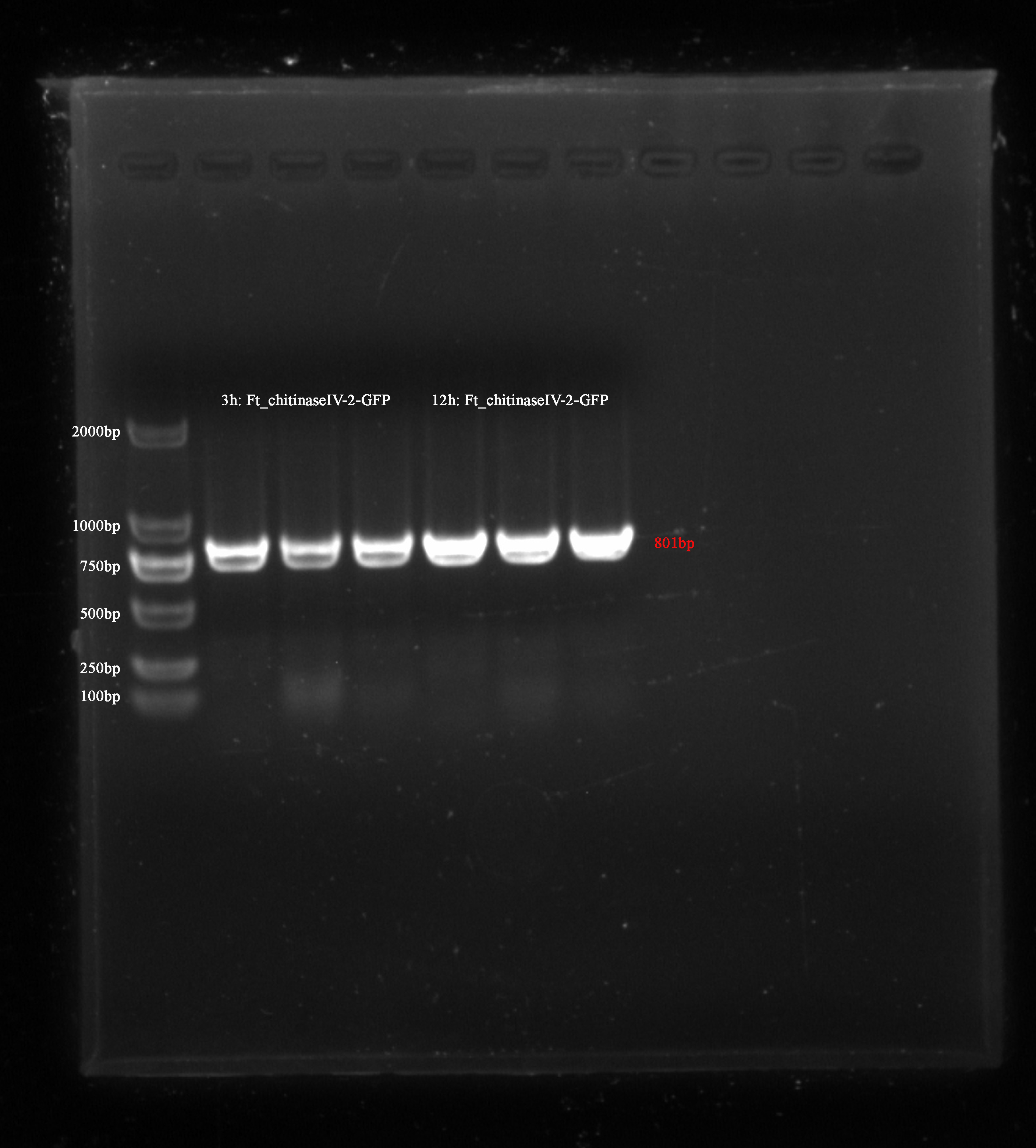

Supplement: Supplementary file 1 — Additional file 1: Figure S1: The original and unprocessed gel image [file 12870_2024_5971_MOESM1_ESM.jpg]
